# Extracts from Report of Deaths in the City of Buffalo, for the Month of August, 1866

**Published:** 1866-09

**Authors:** Sandford Eastman

**Affiliations:** Health Physician


### Extracts from Report of Deaths in the City of Buffalo, for the month of August, 1866.

Whole number of deaths from disease, 146. In addition to the above, 4 stillborn were reported in the city.

Sex.—Males, 89; females, 61. Total, 150.

Nativities.—United States, 108; German States, 17; Ireland, 11; England, 5; France, 1; Canada, 4; unknown, 4.

By whom Certified.—By regular physicians at public institutions, 21; do. in city at large, 70; by irregular practitioners, 22; by coroner, 18; by undertakers, 19. Total, 150.

Causes of Death.—Accident, 4; do. by drowning, 7; apoplexy, cerebral, 3; brain, congestion of, 2; do. softening of, 2; cancer, 1; cholera infantum, 22; cholera morbus, 3; cirrhosis of liver, 2; consumption, 15; convulsions, 5; diarrhœa, 23; disease of the brain, 1; do. of heart, 3; do. of spine, 2; do. of stomach, 1; diphtheria, 1; dropsy, general, 2; do. abdominal, 1; dysentery, 2; epilepsy, 1; fever, typhoid, 7; hæmorrhage from uterus, 1; inflammation of brain and meninges, 7; inflammation of lungs, 4; do. of stomach, 1; do. of womb, 1; intemperance, 1; jaundice, 1; marasmus, 5; measles, 2; murder, 2; obstruction of bowels, 1; old age, 4; paralysis, 1; small pox, 1; syphilis, 2; ulceration of bowels, 1; unknown, 1. Total deaths from disease, 146. Still-born, 3.

Mortality from cholera infantum in August, 1855, was 30; mortality from cholera morbus, 7; mortality from diarrhœa, 84.

The following shows the number of deaths in the city in each month of the present year; the number in 1865, and the average of each month for the five years, 1861 to 1865, inclusive:

| | 1865. | 1866. | 5 y'rs av. |
|---|---|---|---|
| January, | 138 | 127 | 132 |
| February, | 143 | 132 | 122 |
| March, | 113 | 100 | 132 |
| April, | 121 | 123 | 126 |
| May, | 136 | 127 | 127 |
| June, | 116 | 105 | 119 |
| July, | 187 | 138 | 164 |
| August, | 250 | 218 | 224 |

The number of deaths in the first eight months of the present year is 170 less than in the corresponding period of last year, and 144 less than the average for five years. SANDFORD EASTMAN, M. D., Health Physician.

